# 4-[(4-Amino-3-pyrid­yl)imino­meth­yl]benzonitrile

**DOI:** 10.1107/S1600536808037070

**Published:** 2008-11-13

**Authors:** Hoong-Kun Fun, Hadi Kargar, Reza Kia

**Affiliations:** a X-ray Crystallography Unit, School of Physics, Universiti Sains Malaysia, 11800 USM, Penang, Malaysia; b Department of Chemistry, School of Science, Payame Noor University (PNU), Ardakan, Yazd, Iran

## Abstract

The asymmetric unit of the potential mono-Schiff base ligand title compound, C_13_H_10_N_4_, contains two crystallographically independent mol­ecules, *A* and *B*. In mol­ecule *A*, the two rings are twisted from each other by 13.90 (18)°. By contrast, the dihedral angle between the two rings in mol­ecule *B* is 0.67 (19)°. In the crystal structure, mol­ecules are linked through inter­molecular N—H⋯N inter­actions *via R_4_^4^(32)* motifs, forming two-dimensional arrays. The short distances between the centroids of the six-membered rings indicate the existence of π–π inter­actions [centroid–centroid distances = 3.6880 (17)–3.7466 (15) Å].

## Related literature

For details of hydrogen-bond motifs, see: Bernstein *et al.* (1995 ▶). For related structures, see: Li *et al.* (2005 ▶); Bomfim *et al.* (2005 ▶); Glidewell *et al.* (2005 ▶, 2006 ▶); Sun *et al.* (2004 ▶); Fun *et al.* (2008 ▶).
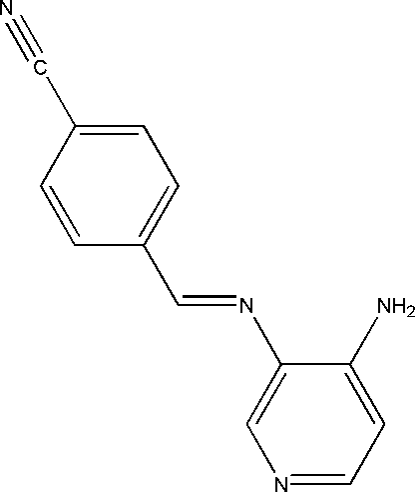


## Experimental

### 

#### Crystal data

C_13_H_10_N_4_
*M* *_r_* = 222.25
Monoclinic, 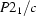

*a* = 13.5560 (8) Å
*b* = 12.3000 (7) Å
*c* = 15.7514 (8) Å
β = 124.651 (2)°
*V* = 2160.5 (2) Å^3^
*Z* = 8
Mo *K*α radiation
μ = 0.09 mm^−1^
*T* = 100.0 (1) K
0.45 × 0.09 × 0.07 mm

#### Data collection

Bruker SMART APEXII CCD area-detector diffractometer
Absorption correction: multi-scan (**SADABS**; Bruker, 2005 ▶) *T*_min_ = 0.962, *T*_max_ = 0.994
18251 measured reflections
3810 independent reflections
2372 reflections with *I* > 2σ(*I*)
*R*_int_ = 0.060

#### Refinement

*R*[*F* ^2^ > 2σ(*F* ^2^)] = 0.074
*wR*(*F* ^2^) = 0.219
*S* = 1.03
3810 reflections
323 parameters
H atoms treated by a mixture of independent and constrained refinement
Δρ_max_ = 0.63 e Å^−3^
Δρ_min_ = −0.29 e Å^−3^

### 

Data collection: *APEX2* (Bruker, 2005 ▶); cell refinement: *APEX2*; data reduction: *SAINT* (Bruker, 2005 ▶); program(s) used to solve structure: *SIR2004* (Burla *et al.*, 2003 ▶); program(s) used to refine structure: *SHELXTL* (Sheldrick, 2008 ▶); molecular graphics: *SHELXTL*; software used to prepare material for publication: *SHELXTL* and *PLATON* (Spek, 2003 ▶).

## Supplementary Material

Crystal structure: contains datablocks global, I. DOI: 10.1107/S1600536808037070/tk2327sup1.cif

Structure factors: contains datablocks I. DOI: 10.1107/S1600536808037070/tk2327Isup2.hkl

Additional supplementary materials:  crystallographic information; 3D view; checkCIF report

## Figures and Tables

**Table 1:** Hydrogen-bond geometry (Å, °)

*D*—H⋯*A*	*D*—H	H⋯*A*	*D*⋯*A*	*D*—H⋯*A*
N3*A*—H2*NA*⋯N4*A*^i^	0.92 (4)	2.26 (4)	3.155 (4)	165 (3)
N3*A*—H1*NA*⋯N1*B*^ii^	0.89 (4)	2.33 (5)	3.080 (4)	143 (4)
N3*B*—H2*NB*⋯N1*A*^iii^	0.89 (4)	2.42 (4)	3.112 (4)	136 (4)
N3*B*—H1*NB*⋯N4*B*^i^	0.90 (4)	2.36 (4)	3.220 (4)	159 (2)

Symmetry codes: (i) 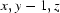; (ii) 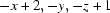; (iii) 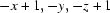.

**Fig. 1.**
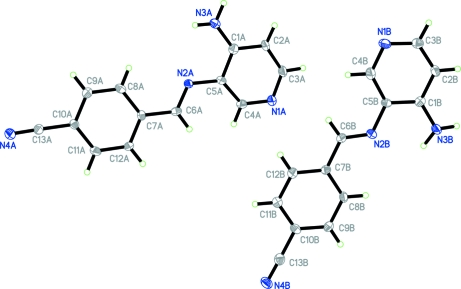
The molecular structure of (I) with atom labels and 50% displacement ellipsoids for non-H atoms.

**Fig. 2.**
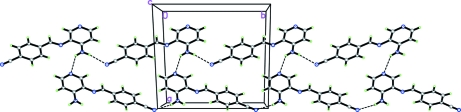
The crystal packing of (I), viewed down the c-axis showing a part of the 2-D array and R44(32) ring motifs. Intermolecular hydrogen bonds are shown as dashed lines.

## supplementary crystallographic information

### Comment

Schiff bases are one of most prevalent mixed-donor ligands in the field of coordination chemistry and play an important role in the development of coordination chemistry (Fun et al.,  2008). Structures of Schiff bases derived from substituted benzaldehydes have been reported previously (Li et al., 2005; Bomfim et al., 2005; Glidewell et al., 2005, 2006; Sun et al., 2004; Fun et al., 2008).

Each imino (C ═N) functional group is co-planar with the adjacent benzene ring in (I), Fig. 1. Two independent molecules, A and B, comprise the crystallographic asymmetric unit. In molecule A, the two phenyl rings are twisted from each other by 13.90 (18)°. The dihedral angle between the two phenyl rings in molecule B is 0.67 (19)° which indicates the molecule is planar. In the crystal structure, molecules are linked together through intermolecular N—H···N interactions via R_4_^4^(32) motifs to form 2-D arrays parallel to the ab-plane, Fig. 2 & Table 1. The short distances between the centroids of the six-membered rings prove the existence of π–π interactions [Cg1···Cg4^i^ = 3.7466 (15) Å, (i) x, 1/2 - y, -1/2 + z; Cg2···Cg3^i^ = 3.6894 (14) Å; Cg3···Cg4^ii^, (ii) 1 - x,-y, 1 - z; Cg1, Cg2, Cg3, and Cg4 are the centroids of the N1A/C1A–C5A, C7A–C12A, N1B/C1B–C5B, and C7B–C12B rings, respectively.

### Experimental

The synthetic method has been described earlier (Fun *et al.*, 2008). Single crystals suitable for X-ray diffraction were obtained by evaporation of an ethanol solution of (I) held at room temperature.

### Refinement

The hydrogen atoms of the amino groups were located from the difference Fourier map and refined freely. The remaining hydrogen atoms were positioned geometrically and refined using a riding model with C—H = 0.93 Å, and with *U*_iso_(H) = 1.2 *U*_eq_ (H).

### Crystal data

**Table:**

C_13_H_10_N_4_	*F*_000_ = 928
*M**_r_* = 222.25	*D*_x_ = 1.367 Mg m^−^^3^
Monoclinic, *P*2_1_/*c*	Mo *K*α radiation λ = 0.71073 Å
Hall symbol: -P 2ybc	Cell parameters from 1699 reflections
*a* = 13.5560 (8) Å	θ = 2.3–30.1º
*b* = 12.3000 (7) Å	µ = 0.09 mm^−^^1^
*c* = 15.7514 (8) Å	*T* = 100 (1) K
β = 124.651 (2)º	Needle, yellow
*V* = 2160.5 (2) Å^3^	0.45 × 0.09 × 0.07 mm
*Z* = 8	

### Data collection

**Table:**

Bruker SMART APEXII CCD area-detector diffractometer	3810 independent reflections
Radiation source: fine-focus sealed tube	2372 reflections with *I* > 2σ(*I*)
Monochromator: graphite	*R*_int_ = 0.060
*T* = 100(1) K	θ_max_ = 25.0º
φ and ω scans	θ_min_ = 1.8º
Absorption correction: multi-scan(*SADABS*; Bruker, 2005)	*h* = −16→16
*T*_min_ = 0.962, *T*_max_ = 0.994	*k* = −14→13
18251 measured reflections	*l* = −18→18

### Refinement

**Table:**

Refinement on *F*^2^	Secondary atom site location: difference Fourier map
Least-squares matrix: full	Hydrogen site location: inferred from neighbouring sites
*R*[*F*^2^ > 2σ(*F*^2^)] = 0.074	H atoms treated by a mixture of independent and constrained refinement
*wR*(*F*^2^) = 0.219	*w* = 1/[σ^2^(*F*_o_^2^) + (0.1298*P*)^2^ + 0.1507*P*] where *P* = (*F*_o_^2^ + 2*F*_c_^2^)/3
*S* = 1.03	(Δ/σ)_max_ < 0.001
3810 reflections	Δρ_max_ = 0.63 e Å^−^^3^
323 parameters	Δρ_min_ = −0.29 e Å^−^^3^
Primary atom site location: structure-invariant direct methods	Extinction correction: none

### Special details

**Table:**

Experimental. The low-temperature data was collected with the Oxford Cyrosystem Cobra low-temperature attachment.
Geometry. All e.s.d.'s (except the e.s.d. in the dihedral angle between two l.s. planes) are estimated using the full covariance matrix. The cell e.s.d.'s are taken into account individually in the estimation of e.s.d.'s in distances, angles and torsion angles; correlations between e.s.d.'s in cell parameters are only used when they are defined by crystal symmetry. An approximate (isotropic) treatment of cell e.s.d.'s is used for estimating e.s.d.'s involving l.s. planes.
Refinement. Refinement of *F*^2^ against ALL reflections. The weighted *R*-factor *wR* and goodness of fit *S* are based on *F*^2^, conventional *R*-factors *R* are based on *F*, with *F* set to zero for negative *F*^2^. The threshold expression of *F*^2^ > σ(*F*^2^) is used only for calculating *R*-factors(gt) *etc*. and is not relevant to the choice of reflections for refinement. *R*-factors based on *F*^2^ are statistically about twice as large as those based on *F*, and *R*- factors based on ALL data will be even larger.

### Fractional atomic coordinates and isotropic or equivalent isotropic displacement parameters (Å^2^)

**Table:**

	*x*	*y*	*z*	*U*_iso_*/*U*_eq_	
N1A	0.6639 (2)	0.12555 (19)	0.38263 (17)	0.0217 (6)	
N2A	0.85668 (19)	0.33048 (19)	0.36498 (16)	0.0148 (6)	
N3A	0.9556 (2)	0.1387 (2)	0.3572 (2)	0.0277 (7)	
N4A	1.0578 (2)	0.9061 (2)	0.36363 (17)	0.0227 (6)	
C1A	0.8618 (2)	0.1349 (2)	0.36579 (19)	0.0185 (7)	
C2A	0.8126 (2)	0.0361 (2)	0.36880 (19)	0.0200 (7)	
H2AA	0.8447	−0.0292	0.3654	0.024*	
C3A	0.7163 (2)	0.0359 (2)	0.3769 (2)	0.0210 (7)	
H3AA	0.6856	−0.0312	0.3784	0.025*	
C4A	0.7119 (2)	0.2198 (2)	0.37969 (19)	0.0184 (7)	
H4AA	0.6773	0.2833	0.3834	0.022*	
C5A	0.8084 (2)	0.2316 (2)	0.37159 (19)	0.0156 (6)	
C6A	0.8305 (2)	0.4207 (2)	0.38827 (19)	0.0173 (7)	
H6AA	0.7799	0.4207	0.4104	0.021*	
C7A	0.8790 (2)	0.5238 (2)	0.38050 (18)	0.0140 (6)	
C8A	0.9575 (2)	0.5272 (2)	0.34913 (18)	0.0189 (7)	
H8AA	0.9781	0.4632	0.3313	0.023*	
C9A	1.0037 (2)	0.6245 (2)	0.34472 (19)	0.0203 (7)	
H9AA	1.0551	0.6263	0.3234	0.024*	
C10A	0.9739 (2)	0.7212 (2)	0.37224 (19)	0.0156 (6)	
C11A	0.8961 (2)	0.7180 (2)	0.40366 (19)	0.0185 (7)	
H11A	0.8755	0.7819	0.4217	0.022*	
C12A	0.8499 (2)	0.6198 (2)	0.40791 (19)	0.0168 (7)	
H12A	0.7987	0.6179	0.4294	0.020*	
C13A	1.0218 (2)	0.8236 (2)	0.36773 (19)	0.0173 (6)	
C3B	0.7831 (2)	−0.3515 (2)	0.62159 (19)	0.0202 (7)	
H3BA	0.8150	−0.4183	0.6211	0.024*	
N2B	0.63747 (19)	−0.05867 (19)	0.62738 (16)	0.0183 (6)	
N3B	0.5661 (2)	−0.2465 (2)	0.66987 (19)	0.0236 (6)	
N4B	0.4350 (2)	0.5212 (2)	0.62229 (18)	0.0269 (6)	
C1B	0.6482 (2)	−0.2527 (2)	0.64597 (19)	0.0160 (7)	
C2B	0.6988 (2)	−0.3504 (2)	0.6444 (2)	0.0191 (7)	
H2BA	0.6757	−0.4152	0.6588	0.023*	
N1B	0.8223 (2)	−0.26157 (19)	0.59977 (17)	0.0225 (6)	
C4B	0.7739 (3)	−0.1680 (2)	0.6022 (2)	0.0232 (7)	
H4BA	0.7994	−0.1047	0.5878	0.028*	
C5B	0.6885 (2)	−0.1573 (2)	0.62439 (19)	0.0153 (6)	
C6B	0.6661 (2)	0.0318 (2)	0.6087 (2)	0.0207 (7)	
H6BA	0.7215	0.0325	0.5916	0.025*	
C7B	0.6158 (2)	0.1352 (2)	0.61273 (19)	0.0156 (6)	
C8B	0.5309 (2)	0.1391 (2)	0.63648 (19)	0.0177 (7)	
H8BA	0.5058	0.0751	0.6501	0.021*	
C9B	0.4837 (3)	0.2381 (2)	0.6398 (2)	0.0197 (7)	
H9BA	0.4278	0.2407	0.6562	0.024*	
C10B	0.5212 (2)	0.3334 (2)	0.6183 (2)	0.0183 (7)	
C11B	0.6066 (3)	0.3310 (2)	0.5956 (2)	0.0219 (7)	
H11B	0.6321	0.3950	0.5823	0.026*	
C12B	0.6525 (3)	0.2323 (2)	0.5929 (2)	0.0242 (7)	
H12B	0.7094	0.2303	0.5776	0.029*	
C13B	0.4722 (3)	0.4381 (3)	0.6207 (2)	0.0222 (7)	
H2NA	0.999 (3)	0.077 (3)	0.366 (2)	0.028 (9)*	
H1NA	0.996 (3)	0.199 (3)	0.365 (3)	0.048 (12)*	
H2NB	0.527 (3)	−0.184 (3)	0.654 (2)	0.044 (11)*	
H1NB	0.538 (3)	−0.311 (3)	0.675 (2)	0.039 (10)*	

### Atomic displacement parameters (Å^2^)

**Table:**

	*U*^11^	*U*^22^	*U*^33^	*U*^12^	*U*^13^	*U*^23^
N1A	0.0241 (14)	0.0159 (14)	0.0297 (13)	−0.0010 (11)	0.0180 (12)	0.0011 (11)
N2A	0.0128 (12)	0.0135 (14)	0.0182 (11)	0.0012 (10)	0.0089 (10)	0.0005 (9)
N3A	0.0293 (16)	0.0154 (17)	0.0494 (17)	0.0009 (13)	0.0290 (14)	−0.0006 (13)
N4A	0.0240 (14)	0.0166 (15)	0.0305 (13)	0.0020 (11)	0.0173 (12)	0.0021 (11)
C1A	0.0197 (15)	0.0196 (18)	0.0157 (13)	−0.0026 (12)	0.0097 (12)	−0.0022 (12)
C2A	0.0239 (16)	0.0113 (17)	0.0263 (15)	−0.0020 (13)	0.0152 (13)	0.0005 (12)
C3A	0.0225 (16)	0.0148 (17)	0.0260 (15)	−0.0034 (13)	0.0140 (13)	0.0002 (12)
C4A	0.0154 (15)	0.0197 (18)	0.0203 (14)	0.0019 (12)	0.0103 (13)	−0.0010 (12)
C5A	0.0153 (14)	0.0134 (16)	0.0165 (13)	0.0020 (12)	0.0082 (12)	−0.0009 (11)
C6A	0.0141 (14)	0.0202 (18)	0.0179 (13)	0.0025 (12)	0.0094 (12)	−0.0004 (12)
C7A	0.0119 (13)	0.0140 (16)	0.0130 (12)	−0.0016 (11)	0.0053 (11)	0.0002 (11)
C8A	0.0216 (15)	0.0155 (17)	0.0191 (14)	0.0006 (12)	0.0113 (13)	−0.0034 (12)
C9A	0.0213 (16)	0.0210 (18)	0.0226 (14)	−0.0014 (13)	0.0149 (13)	−0.0006 (12)
C10A	0.0147 (14)	0.0136 (16)	0.0161 (13)	0.0008 (12)	0.0073 (12)	0.0008 (11)
C11A	0.0171 (15)	0.0163 (17)	0.0209 (14)	0.0033 (12)	0.0101 (13)	−0.0015 (12)
C12A	0.0151 (14)	0.0175 (17)	0.0209 (14)	0.0008 (12)	0.0121 (12)	−0.0004 (12)
C13A	0.0169 (15)	0.0178 (17)	0.0178 (14)	0.0000 (13)	0.0102 (12)	0.0006 (12)
C3B	0.0184 (15)	0.0199 (18)	0.0216 (15)	−0.0007 (12)	0.0109 (13)	−0.0029 (12)
N2B	0.0195 (13)	0.0145 (15)	0.0220 (12)	0.0044 (10)	0.0124 (11)	0.0027 (10)
N3B	0.0266 (15)	0.0137 (17)	0.0392 (15)	0.0009 (13)	0.0239 (13)	0.0023 (12)
N4B	0.0262 (14)	0.0168 (16)	0.0405 (15)	0.0033 (12)	0.0206 (13)	0.0012 (12)
C1B	0.0094 (13)	0.0216 (18)	0.0130 (13)	−0.0003 (12)	0.0040 (11)	0.0003 (11)
C2B	0.0182 (15)	0.0154 (17)	0.0218 (14)	0.0008 (12)	0.0103 (13)	0.0033 (12)
N1B	0.0213 (14)	0.0181 (15)	0.0310 (13)	0.0015 (11)	0.0166 (12)	−0.0030 (11)
C4B	0.0260 (16)	0.0171 (18)	0.0287 (15)	−0.0014 (13)	0.0169 (14)	0.0023 (13)
C5B	0.0126 (14)	0.0164 (17)	0.0142 (13)	0.0026 (11)	0.0060 (11)	−0.0008 (11)
C6B	0.0220 (15)	0.0198 (18)	0.0285 (15)	0.0034 (13)	0.0193 (13)	0.0038 (13)
C7B	0.0137 (14)	0.0169 (17)	0.0163 (13)	−0.0002 (12)	0.0087 (12)	−0.0007 (11)
C8B	0.0206 (16)	0.0141 (17)	0.0195 (14)	−0.0015 (12)	0.0120 (13)	0.0015 (11)
C9B	0.0192 (15)	0.0211 (18)	0.0238 (14)	−0.0015 (13)	0.0152 (13)	0.0001 (12)
C10B	0.0188 (15)	0.0151 (17)	0.0168 (13)	0.0004 (12)	0.0077 (12)	−0.0021 (11)
C11B	0.0264 (16)	0.0172 (18)	0.0264 (15)	−0.0005 (13)	0.0175 (14)	−0.0001 (12)
C12B	0.0269 (17)	0.0200 (18)	0.0345 (17)	0.0018 (13)	0.0227 (15)	0.0034 (13)
C13B	0.0192 (15)	0.0229 (19)	0.0246 (15)	−0.0025 (14)	0.0124 (13)	0.0000 (13)

### Geometric parameters (Å, °)

**Table:**

N1A—C3A	1.343 (3)	C3B—N1B	1.354 (3)
N1A—C4A	1.343 (3)	C3B—C2B	1.379 (4)
N2A—C6A	1.281 (3)	C3B—H3BA	0.9300
N2A—C5A	1.413 (3)	N2B—C6B	1.267 (3)
N3A—C1A	1.354 (4)	N2B—C5B	1.411 (3)
N3A—H2NA	0.93 (3)	N3B—C1B	1.366 (4)
N3A—H1NA	0.88 (4)	N3B—H2NB	0.88 (4)
N4A—C13A	1.143 (3)	N3B—H1NB	0.90 (4)
C1A—C2A	1.399 (4)	N4B—C13B	1.147 (4)
C1A—C5A	1.422 (4)	C1B—C2B	1.391 (4)
C2A—C3A	1.381 (4)	C1B—C5B	1.415 (4)
C2A—H2AA	0.9300	C2B—H2BA	0.9300
C3A—H3AA	0.9300	N1B—C4B	1.336 (4)
C4A—C5A	1.392 (4)	C4B—C5B	1.393 (4)
C4A—H4AA	0.9300	C4B—H4BA	0.9300
C6A—C7A	1.466 (4)	C6B—C7B	1.461 (4)
C6A—H6AA	0.9300	C6B—H6BA	0.9300
C7A—C12A	1.389 (4)	C7B—C12B	1.396 (4)
C7A—C8A	1.406 (4)	C7B—C8B	1.399 (4)
C8A—C9A	1.370 (4)	C8B—C9B	1.390 (4)
C8A—H8AA	0.9300	C8B—H8BA	0.9300
C9A—C10A	1.402 (4)	C9B—C10B	1.394 (4)
C9A—H9AA	0.9300	C9B—H9BA	0.9300
C10A—C11A	1.398 (4)	C10B—C11B	1.393 (4)
C10A—C13A	1.437 (4)	C10B—C13B	1.458 (4)
C11A—C12A	1.379 (4)	C11B—C12B	1.375 (4)
C11A—H11A	0.9300	C11B—H11B	0.9300
C12A—H12A	0.9300	C12B—H12B	0.9300
			
C3A—N1A—C4A	114.9 (2)	N1B—C3B—C2B	124.1 (3)
C6A—N2A—C5A	120.6 (2)	N1B—C3B—H3BA	117.9
C1A—N3A—H2NA	121.2 (19)	C2B—C3B—H3BA	117.9
C1A—N3A—H1NA	123 (2)	C6B—N2B—C5B	121.8 (2)
H2NA—N3A—H1NA	112 (3)	C1B—N3B—H2NB	115 (2)
N3A—C1A—C2A	121.7 (3)	C1B—N3B—H1NB	115 (2)
N3A—C1A—C5A	121.2 (3)	H2NB—N3B—H1NB	125 (3)
C2A—C1A—C5A	117.0 (3)	N3B—C1B—C2B	122.7 (3)
C3A—C2A—C1A	119.9 (3)	N3B—C1B—C5B	120.4 (3)
C3A—C2A—H2AA	120.0	C2B—C1B—C5B	116.8 (3)
C1A—C2A—H2AA	120.0	C3B—C2B—C1B	120.2 (3)
N1A—C3A—C2A	124.6 (3)	C3B—C2B—H2BA	119.9
N1A—C3A—H3AA	117.7	C1B—C2B—H2BA	119.9
C2A—C3A—H3AA	117.7	C4B—N1B—C3B	115.3 (3)
N1A—C4A—C5A	126.3 (3)	N1B—C4B—C5B	125.4 (3)
N1A—C4A—H4AA	116.9	N1B—C4B—H4BA	117.3
C5A—C4A—H4AA	116.9	C5B—C4B—H4BA	117.3
C4A—C5A—N2A	126.5 (3)	C4B—C5B—N2B	125.6 (3)
C4A—C5A—C1A	117.2 (3)	C4B—C5B—C1B	118.2 (3)
N2A—C5A—C1A	116.2 (2)	N2B—C5B—C1B	116.2 (2)
N2A—C6A—C7A	121.0 (2)	N2B—C6B—C7B	122.8 (3)
N2A—C6A—H6AA	119.5	N2B—C6B—H6BA	118.6
C7A—C6A—H6AA	119.5	C7B—C6B—H6BA	118.6
C12A—C7A—C8A	119.3 (3)	C12B—C7B—C8B	118.8 (3)
C12A—C7A—C6A	119.3 (2)	C12B—C7B—C6B	120.0 (2)
C8A—C7A—C6A	121.3 (3)	C8B—C7B—C6B	121.2 (3)
C9A—C8A—C7A	120.2 (3)	C9B—C8B—C7B	120.4 (3)
C9A—C8A—H8AA	119.9	C9B—C8B—H8BA	119.8
C7A—C8A—H8AA	119.9	C7B—C8B—H8BA	119.8
C8A—C9A—C10A	120.3 (3)	C8B—C9B—C10B	119.2 (3)
C8A—C9A—H9AA	119.8	C8B—C9B—H9BA	120.4
C10A—C9A—H9AA	119.8	C10B—C9B—H9BA	120.4
C11A—C10A—C9A	119.6 (3)	C11B—C10B—C9B	121.0 (3)
C11A—C10A—C13A	119.7 (3)	C11B—C10B—C13B	118.9 (3)
C9A—C10A—C13A	120.7 (3)	C9B—C10B—C13B	120.1 (3)
C12A—C11A—C10A	119.8 (3)	C12B—C11B—C10B	118.9 (3)
C12A—C11A—H11A	120.1	C12B—C11B—H11B	120.5
C10A—C11A—H11A	120.1	C10B—C11B—H11B	120.5
C11A—C12A—C7A	120.8 (3)	C11B—C12B—C7B	121.6 (3)
C11A—C12A—H12A	119.6	C11B—C12B—H12B	119.2
C7A—C12A—H12A	119.6	C7B—C12B—H12B	119.2
N4A—C13A—C10A	178.6 (3)	N4B—C13B—C10B	178.8 (3)
			
N3A—C1A—C2A—C3A	−179.5 (3)	N1B—C3B—C2B—C1B	0.1 (4)
C5A—C1A—C2A—C3A	0.1 (4)	N3B—C1B—C2B—C3B	−178.8 (2)
C4A—N1A—C3A—C2A	0.2 (4)	C5B—C1B—C2B—C3B	−0.7 (4)
C1A—C2A—C3A—N1A	−0.3 (4)	C2B—C3B—N1B—C4B	0.4 (4)
C3A—N1A—C4A—C5A	−0.1 (4)	C3B—N1B—C4B—C5B	−0.3 (4)
N1A—C4A—C5A—N2A	177.7 (2)	N1B—C4B—C5B—N2B	179.8 (2)
N1A—C4A—C5A—C1A	0.0 (4)	N1B—C4B—C5B—C1B	−0.3 (4)
C6A—N2A—C5A—C4A	15.3 (4)	C6B—N2B—C5B—C4B	0.4 (4)
C6A—N2A—C5A—C1A	−167.0 (2)	C6B—N2B—C5B—C1B	−179.5 (2)
N3A—C1A—C5A—C4A	179.6 (2)	N3B—C1B—C5B—C4B	178.9 (2)
C2A—C1A—C5A—C4A	−0.1 (3)	C2B—C1B—C5B—C4B	0.7 (4)
N3A—C1A—C5A—N2A	1.7 (4)	N3B—C1B—C5B—N2B	−1.1 (4)
C2A—C1A—C5A—N2A	−178.0 (2)	C2B—C1B—C5B—N2B	−179.3 (2)
C5A—N2A—C6A—C7A	−179.3 (2)	C5B—N2B—C6B—C7B	−179.1 (2)
N2A—C6A—C7A—C12A	−179.0 (2)	N2B—C6B—C7B—C12B	178.9 (3)
N2A—C6A—C7A—C8A	−1.2 (4)	N2B—C6B—C7B—C8B	−0.9 (4)
C12A—C7A—C8A—C9A	−0.7 (4)	C12B—C7B—C8B—C9B	0.3 (4)
C6A—C7A—C8A—C9A	−178.5 (2)	C6B—C7B—C8B—C9B	−179.9 (2)
C7A—C8A—C9A—C10A	0.5 (4)	C7B—C8B—C9B—C10B	0.6 (4)
C8A—C9A—C10A—C11A	−0.4 (4)	C8B—C9B—C10B—C11B	−1.2 (4)
C8A—C9A—C10A—C13A	−179.9 (2)	C8B—C9B—C10B—C13B	179.5 (2)
C9A—C10A—C11A—C12A	0.4 (4)	C9B—C10B—C11B—C12B	1.0 (4)
C13A—C10A—C11A—C12A	179.9 (2)	C13B—C10B—C11B—C12B	−179.7 (2)
C10A—C11A—C12A—C7A	−0.5 (4)	C10B—C11B—C12B—C7B	−0.1 (4)
C8A—C7A—C12A—C11A	0.7 (4)	C8B—C7B—C12B—C11B	−0.5 (4)
C6A—C7A—C12A—C11A	178.6 (2)	C6B—C7B—C12B—C11B	179.7 (3)

### Hydrogen-bond geometry (Å, °)

**Table:**

*D*—H···*A*	*D*—H	H···*A*	*D*···*A*	*D*—H···*A*
N3A—H2NA···N4A^i^	0.92 (4)	2.26 (4)	3.155 (4)	165 (3)
N3A—H1NA···N1B^ii^	0.89 (4)	2.33 (5)	3.080 (4)	143 (4)
N3B—H2NB···N1A^iii^	0.89 (4)	2.42 (4)	3.112 (4)	136 (4)
N3B—H1NB···N4B^i^	0.90 (4)	2.36 (4)	3.220 (4)	159 (2)

Symmetry codes: (i) *x*, *y*−1, *z*; (ii) −*x*+2, −*y*, −*z*+1; (iii) −*x*+1, −*y*, −*z*+1.
